# Regulation of Eye Determination and Regionalization in the Spider *Parasteatoda tepidariorum*

**DOI:** 10.3390/cells11040631

**Published:** 2022-02-11

**Authors:** Luis Baudouin-Gonzalez, Amber Harper, Alistair P. McGregor, Lauren Sumner-Rooney

**Affiliations:** 1 Oxford University Museum of Natural History, University of Oxford, Oxford OX1 3PW, UK; 2 Department of Biological and Medical Sciences, Oxford Brookes University, Oxford OX3 0BP, UK; 19012072@brookes.ac.uk (A.H.); amcgregor@brookes.ac.uk (A.P.M.); 3 Department of Biosciences, Durham University, Durham DH1 3LE, UK; 4 Museum für Naturkunde, Leibniz Institute for Biodiversity and Evolution, 10115 Berlin, Germany; lauren.sumner-rooney@mfn.berlin

**Keywords:** eye development, spiders, Wnt, Pax6, retinal determination genes

## Abstract

Animal visual systems are enormously diverse, but their development appears to be controlled by a set of conserved retinal determination genes (RDGs). Spiders are particular masters of visual system innovation, and offer an excellent opportunity to study the evolution of animal eyes. Several RDGs have been identified in spider eye primordia, but their interactions and regulation remain unclear. From our knowledge of RDG network regulation in *Drosophila melanogaster*, we hypothesize that orthologs of *Pax6*, *eyegone*, Wnt genes, *hh*, *dpp*, and *atonal* could play important roles in controlling eye development in spiders. We analyzed the expression of these genes in developing embryos of the spider *Parasteatoda* *tepidariorum*, both independently and in relation to the eye primordia, marked using probes for the RDG *sine oculis*. Our results support conserved roles for Wnt genes in restricting the size and position of the eye field, as well as for *atonal* initiating photoreceptor differentiation. However, we found no strong evidence for an upstream role of *Pax6* in eye development, despite its label as a master regulator of animal eye development; nor do *eyg*, *hh* or *dpp* compensate for the absence of *Pax6*. Conversely, our results indicate that *hh* may work with Wnt signaling to restrict eye growth, a role similar to that of *Sonic* *hedgehog* (*Shh*) in vertebrates.

## 1. Introduction

**Figure 1:**
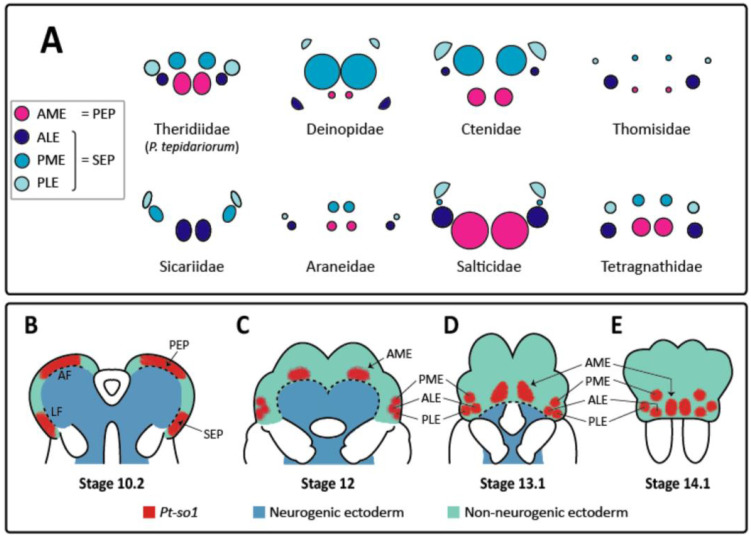
Variation in spider eye morphology and dynamics of eye development in *Parasteatoda tepidariorum*. (**A**) Diagram displaying the highly diverse eye arrangements of eight different spider families. After Morehouse et al. [1]. (**B**–**E**) Dynamics of eye development, as followed by *Pt-so1* expression throughout embryogenesis in *P. tepidariorum* [12]. At stage 10.2 (**B**), *Pt-so1* expression is restricted to the regions of non-neurogenic ectoderm adjacent to the anterior (AF) and lateral (LF) furrows (dashed lines), establishing the principal (PEP) and secondary (SEP) eye primordia. At stage 12 (**C**), *Pt-so1* in the PEP becomes confined to a smaller group of cells and travels, with the overgrowing edge of the non-neurogenic ectoderm (dashed line) towards its final position. *Pt-so1* expression in the SEP splits into three distinct domains corresponding to each secondary eye. In the following stages (**D**), these domains continue migrating towards their final positions in the developing head of *P. tepidariorum* (**E**). AME, anterior median eye; ALE, anterior lateral eye; PME, posterior median eye; PLE, posterior lateral eye.

Spiders have some of the most diverse visual systems in the animal kingdom [1]. They exhibit substantial variation on a conserved anatomical blueprint, including eye size, arrangement, function, and even number (Figure 1A). Visual ability, including spatial resolution, sensitivity, and the detection of polarization and color, varies widely with behavioral and ecological needs [1]. Most spiders have four pairs of eyes, which can be divided into two morphologically and developmentally distinct types: the principal eyes, comprising the anterior median eyes (AMEs), and the secondary eyes, comprising the anterior lateral (ALEs), posterior median (PMEs) and posterior lateral eyes (PLEs) [1,2] (Figure 1). These are likely homologous to structures across Arthropoda, with the principal and secondary eyes being homologous to insect ocelli and compound eyes, respectively [1]. Thus, spiders represent an exceptional model to study the evolution of visual systems and the developmental mechanisms underpinning their variation, both within a highly diverse order and across the largest animal phylum. 

Despite there being more than 40 estimated evolutionary origins of vision [3], studies of eye development across the animal kingdom have consistently found a core set of genes that determine eye fate, including members of the Pax4/6, Six1/2, Six3/6, Eya and Dac gene families [4,5]. There is evidence to suggest that their roles and interactions can also be conserved in retinal determination gene (RDG) networks. The *Pax6* genes, for example, appear to sit near the top of the RDG regulatory network, and are usually required for the activation of conserved downstream genes, such as sine oculis (so; a Six1/2 family gene), dachshund (dac) and eyes absent (eya), in both insects and vertebrates [4,6]. Given both the similarity and the importance of *Pax6* genes’ upstream role in such widely separated phylogenetic groups, it has been widely interpreted as a ‘master regulator’ of eye development [6,7]. Indeed, *Pax6* genes have been implicated in eye development in a wide range of other organisms, including mollusks, annelids, echinoderms, and planarians [8,9,10,11]. Although many other RDGs appear to be conserved between taxa, their specific roles and interactions have not been extensively explored.

In spiders, our understanding of eye development is fairly limited. The two eye types have distinct developmental origins, stemming from the independent regions of non-neural ectoderm at the dorsal and lateral margins of the developing head (Figure 1B–E) [12]. In *Parasteatoda tepidariorum*, these migrate ventrally and medially as the head capsule closes, forming distinct primordia for each eye before invaginating to form eyecups (Figure 1B–E) [2,12]. Several key RDGs have already been detected in spiders: studies of *P. tepidariorum* and *Cupiennius salei* demonstrated the expression of *so*, *eya*, *dac*, *six3* and *otd* orthologues in the developing eyes [12,13], with differences in expression between eye types and between species. However, very little is known about how these RDGs interact or how they are regulated, which is critical to understanding how key features such as eye size and position are controlled. Thanks to the broad conservation of RDG networks, we can look to other arthropod systems to identify candidates for involvement in regulation.

**Figure 2:**
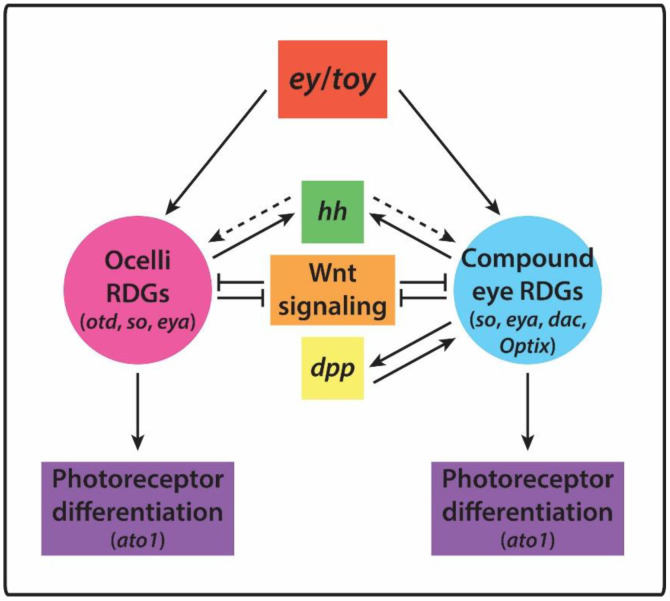
Gene regulatory network controlling eye development in *Drosophila melanogaster*. *Pax6* genes *ey* and *toy* activate RDG networks regulating compound eye (light blue) and ocellus (pink) development. Additional activation occurs via *dpp* and *hh*. These networks are repressed by Wnt signaling, and culminate in the downstream activation of *ato*, which triggers photoreceptor differentiation.

The genetic regulation of arthropod eye development is best understood in *Drosophila melanogaster*. In both the compound eyes and ocelli, a *Pax6* homologue, *twin of eyeless* (*toy*), appears to initiate specification and activates subsequent RDGs, encoding transcription factors [14,15]. These include *so* in both compound eyes and ocelli, and *eya*, *eyeless* (*ey*, another *Pax6* homologue), and *Optix* (a Six3/6 family gene) in the compound eyes (Figure 2) [14,16,17,18]. *Eya* expression is also indirectly activated by *hedgehog* (*hh*) in both the compound eyes and ocelli [19,20,21,22]. In the compound eye, these four transcription factors, together with *dac*, *dpp*, and *hh,* reinforce each other’s expression to initiate and maintain retinal determination [19,20]. These genes ultimately activate the expression of downstream genes involved in eye differentiation. In both the compound eyes and ocelli, *so* and *eya* activate the expression of the transcription factor Atonal (Ato), which plays a key role in photoreceptor cell fate and ommatidium formation (Figure 2) [23,24].

As well as triggering the determination of specific tissues, developmental controls are also required to restrict the primordia in order to produce well-defined organs of the correct size in the correct position. Wnt signaling regulates eye development in highly divergent phyla from planarians [9] to insects and vertebrates [25,26,27]. In *D. melanogaster,* Wnt1/Wingless (wg) regulates cell division in the eye primordia, but plays a critical role in confining the expression of RDGs to the eye field by repressing *so*, *eya*, and *dac* in the surrounding head tissue [20,27]. Wg signaling also inhibits *eya* and *so* expression in the region surrounding the ocellus primordia, but within the primordia, it directly activates *otd* expression, which ultimately leads to the activation of *eya* expression [22].

Combined, these studies in *Drosophila* imply a model wherein *Pax6* homologues initiate cascades of RDGs including *so* and *eya*, with additional genes specific to eye type, that culminate in photoreceptor differentiation mediated by *ato* expression (Figure 2). The spatial distribution of this process, and thus the size and position of the compound eyes and ocelli, is restricted by Wnt signaling. 

This model may be informative of our understanding of eye development in spiders and provides several candidates for the exploration of RDG regulation. Indeed, some similarities to *D. melanogaster* emerge from our existing knowledge of spider eye development and its genetic basis. Most RDG orthologs analyzed are expressed in at least one pair of eyes, including *so*, *otd*, *dac*, and *eya* [12,13]. Recent functional analysis has also demonstrated that the RNAi knockdown of *so* can also cause the loss of eyes in *P. tepidariorum* [28], indicating support for this central part of the RDG network being conserved between spiders and insects. The suites of RDGs specific to eye type are also very similar between *D. melanogaster* and *P. tepidariorum*, with *Optix*/*six3.2* and *dac*/*dac1* being expressed in the compound/secondary eyes and *otd*/*otd2* in the ocelli/principal eyes, respectively [12]. Given the shared evolutionary origins of the eye types, this could reflect an ancestral distinction between them, and thus, another conserved aspect of eye development in lineages separated by more than half a billion years [29].

However, it remains unclear as to what extent the wider eye determination pathway and its regulation are conserved between these two groups. First, despite *Pax6′*s ascribed role as a universal regulator of animal eye development [6,8,30,31,32], there is a lack of clear evidence for such a role in chelicerates [1,12,13]. While one *Pax6* ortholog is expressed in the AMEs of *C. salei* at a late stage of eye development, neither paralog was detected in any of the developing eyes of *P. tepidariorum* [12,13]. Despite this, the downstream machinery, including *eya*, *so*, and *dac*, appears to be more consistent. Previous work on *Pax6* expression, however, only examined embryos from stage 10 onwards, when *so, eya*, and *otd* are already expressed; it may be that initiation via *Pax6* occurs in earlier stages.

Alternatively, their activation may be controlled by another factor or factors. In *D*. *melanogaster*, *dpp* also activates *so*, *eya* and *dac* via *hh*, and *hh* indirectly activates *otd* [19,21,22]. However, neither *dpp* nor *hh* expression has been studied in relation to the development of spider eyes. Furthermore, an additional member of the Pax4/6 gene family was recently identified in *P. tepidariorum*, which appears to be an ortholog of the *D. melanogaster* gene *eye gone* (*eyg*) [33]. *eyg* is required for eye development in *D. melanogaster* and can partially substitute for *Pax6* activity [34,35]. These genes could represent alternative routes for the activation of RDG network components.

Second, in addition to the RDG orthologs, the expression of *ato* orthologs was analysed for *C. salei*, but neither of the two copies identified in this species were found to be expressed in the developing eyes [13]. This is inconsistent with the hypothesis that *ato* is a key downstream actor regulating photoreceptor differentiation in ancestral arthropod eyes [1,23]. However, *ato* expression has not yet been studied in *P. tepidariorum*, the main model species for spider development.

Although the important role of Wg in restricting the eye field is known in *D. melanogaster*, Wnt gene expression has not yet been studied in the context of eye development in spiders. Five Wnt genes are expressed in the developing head of *P. tepidariorum*: *Pt-Wnt2*, *Pt-Wnt5*, *Pt-Wnt7.2*, *Pt-Wnt8*, and *Pt-Wnt16* [36], but their relationships with and/or proximities to eye primordia were not examined. These Wnt genes are good candidates for involvement in the patterning of head structures, possibly including a role in eye development [25]. Eye size and position are key variable traits in spiders, with different families exhibiting extreme enlargement and even loss; the role of Wnt genes in restricting the size and location of the eye field could therefore represent a key mechanism for the evolution of diversity in spider visual systems.

To further investigate the regulation of eye development in *P. tepidariorum*, we performed a detailed expression analysis of three key components in *P. tepidariorum*: *Pax6* (initiation), *ato* (photoreceptor differentiation) and the Wnt genes (suppression). Furthermore, given the uncertainty of *Pax6′*s involvement, we also analyzed the expression of *eyg*, *hh*, and *dpp*.

## 2. Materials and Methods

### 2.1. Parasteatoda tepidariorum Culture and Embryo Fixation

*P. tepidariorum* were maintained at 25 °C with a 12:12 h light:dark cycle. Spiderlings and adult males were fed on a diet of vestigial mutant flies (*Drosophila melanogaster*), and juvenile and adult females on a diet of small crickets (*Gryllodes sigillatus*).

*P. tepidariorum* embryos were staged according to Mittmann and Wolff [37], and fixed following the protocol in Akiyama-Oda et al. [38], with minor modifications: after transferring to methanol, the embryos were left in this solution for at least 30 min at room temperature, followed by overnight incubation at −20 °C. The vitelline membranes of fixed embryos were removed by dissection with Dumont 5 forceps in methanol.

### 2.2. Identification and Phylogenetic Analysis of Atonal Genes

To identify *ato* genes in *P. tepidariorum*, Ato protein sequences previously identified in *C. salei* [13] were used as queries in a BLAST search against the available transcriptome of *P. tepidariorum* [39]. The protein sequence alignments of basic Helix-loop-Helix protein domains of *ato* and *twist* orthologs from *P. tepidariorum*, *C. salei*, *Centruroides sculpturatus, Ixodes scapularis, Strigamia maritima*, *Daphnia magna*, *Tribolium castaneum*, *Apis mellifera* and *Drosophila melanogaster* were generated in MEGA v.7 using the MUSCLE v.3 algorithm (default settings) [40]. To confirm gene identity, we performed a phylogenetic analysis using RAxML-NG v.1.0.2 [41,42] with an LG substitution model, as suggested by ModelTest-NG [43], with nodal support inferred using the rapid bootstrapping algorithm (1000 replicates). The resulting tree was visualized using the FigTree v1.4.4 software (http://tree.bio.ed.ac.uk/software/figtree/, accessed on 1 December 2021).

### 2.3. Cloning and Probe Synthesis

Total RNA was extracted using QiAzol (Qiagen) from stage 1-14 *P. tepidariorum* embryos, and cDNA was generated using the QuantiTect reverse transcription kit (Qiagen). Gene fragments were amplified from cDNA by PCR and cloned into the pCR ^®^ 4-TOPO ^®^ TA vector using the TOPO ^®^ TA cloning kit for sequencing (ThermoFisher Scientific). A list of primers used is provided in Supplementary Materials Table S1. RNA probes were synthesized using T7 (10881775001, Roche) or T3 polymerase (11031163001, Roche), depending on the orientation of the cloned fragment in the pCR ^®^ TOPO ^®^ TA plasmid, with either DIG RNA labeling mix (for colorimetric ISH and dFISH; 11277073910, Roche) or Fluorescein RNA labeling mix (for dFISH; 11685619910, Roche).

### 2.4. In Situ Hybridization (ISH) in P. tepidariorum Embryos

To examine the expression patterns of *ato*, *eyg* and *dpp*, colorimetric ISH was performed following the whole-mount protocol described in Prpic et al. [44], with minor modifications: steps 4–8 were replaced by two 10-min washes in PBS-Tween-20 (0.02%) (PBS-T), and at step 18, the embryos were incubated for 30 min. Embryos were post-fixed before ethanol treatment to decrease background: incubation for 10 min in inactivation buffer (75 g glycine, 600 μL 1N HCl, 50 μL 10% Tween-20 and dH_2_O to 10 mL), followed by three washes in PBS-T, a 5-min wash in 50% ethanol in PBS-T, a quick wash (~30s) in 100% ethanol until background had decreased, a second 5-min wash in 50% ethanol in PBS-T, and finally, two 5-min washes in PBS-T. Embryos were then counterstained with DAPI (1:2000; 10236276001, Roche) for ~20 min and stored in 80% glycerol in PBS at 4 °C. Imaging was performed using a Zeiss Axio Zoom V.16. DAPI overlays were generated in Photoshop CS6.

### 2.5. Double Fluorescent ISH (dFISH) in P. tepidariorum Embryos

To establish the expression patterns of Wnt genes, *Pax6* genes, *ato*, and *hh* relative to *so*, dFISH was performed following a protocol modified from Clark and Akam [45]. Embryos were gradually moved from methanol to PBS-T and then washed twice for 15 min in PBS-T. Embryos were then transferred to hybridization buffer, hybridized overnight at 65 °C, and washed post-hybridization, as detailed in Prpic et al. [44]. Moreover, 2 μL of each probe (DIG- and FITC-labelled) were used in the hybridization step. Incubation in AP-conjugated anti-DIG (1:2000; 11093274910, Roche) and POD-conjugated anti-FITC (1:2000; 11426346910, Roche) was carried out for two hours, following 30 min incubation in 1× Blocking solution. Tyramide biotin amplification (TSA Plus Biotin Kit, NEL749A001KT, Perkin Elmer) was performed for 10 min, followed by incubation for 90 min in streptavidin Alexa Fluor 488 conjugate (1:500; S11223, ThermoFisher Scientific). AP signal was visualized by Fast Red staining (1/2 Fast Red tablet; 4210, Kem En Tec Diagnostics, Taastrup, Denmark). Counterstaining with DAPI (1:2000; 10236276001, Roche) was carried out for 5–10 min.

The head regions of stained embryos were dissected and flat-mounted prior to imaging. Imaging was performed using a Zeiss LSM800 confocal with Airyscan. Images were processed using the FIJI software [46].

## 3. Results

### 3.1. Expression of Pax6 Orthologs during Eye Development of P. tepidariorum

**Figure 3:**
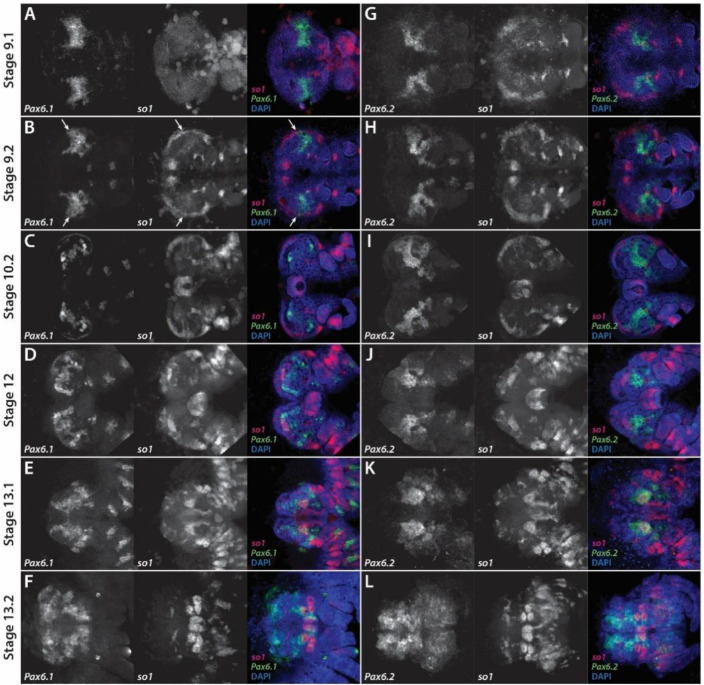
Expression of *Pax6* genes in the developing head of *Parasteatoda tepidariorum* embryos. (**A**–**F**) Expression of *Pt-Pax6.1* in the developing head of stage 9.1 (**A**), 9.2 (**B**), 10.2 (**C**), 12 (**D**), 13.1 (**E**) and 13.2 (**F**) *P. tepidariorum* embryos, co-stained for *Pt-so1* to visualise eye development. *Pt-Pax6.1* expression only overlaps that of *Pt-so1* at stage 9.2 (**B**), in a small region in the lateral edges of the head lobes (white arrows). In all other stages, *Pt-Pax6.1* expression is restricted to the developing brain (**C**–**F**). (**G**–**L**) Expression of *Pt-Pax6.2* in the developing head of stage 9.1 (**G**), 9.2 (**H**), 10.2 (**I**), 12 (**J**), 13.1 (**K**) and 13.2 (**L**) *P. tepidariorum* embryos. *Pt-Pax6.2* expression never overlaps that of *Pt-so1* in the eye primordia and is restricted to the developing brain. (**G**–**L**). Anterior is to the left in all images.

To address whether *Pax6* genes could play a role in spider eye development, we performed a detailed expression pattern analysis of the two *Pax6* paralogs of *P. tepidariorum*, using *Pt-so1* as a marker of the developing eye primordia. Previous work described *Pt-so1* expression from stage 10.2 onwards [12]; however, we occasionally observed *Pt-so1* expression in the developing head of late stage 9.1 embryos (e.g., Figure 3G), and in all embryos throughout stage 9.2 (Figure 3B,H). Where *Pt-so1* expression was present at stage 9.1, it appeared along the lateral extremes of the developing head, in the non-neurogenic ectoderm. Expression is split into two domains, corresponding to the positions of the principal and secondary eye primordia, but is quite diffuse. At stage 9.2, *Pt-so1* expression in these domains is stronger and more clearly defined, being restricted to the very lateral edges (Figure 3B,H).

At stage 9.1, *Pt-Pax6.1* expression is restricted to a pair of large domains in the head primordia, extending from the lateral edges towards the center (Figure 3A). A similar pattern of *Pt-Pax6.1* expression is visible at stage 9.2, which slightly overlaps with the center of *Pt-so1* expression domains at the lateral edges of the developing head (Figure 3B, arrows). At stage 10.2, *Pt-Pax6.1* expression appears to be restricted to the neuroectoderm, in a region between the anterior and lateral furrows (Figure 3C). This appears to be adjacent to, instead of overlapping, *Pt-so1* expression, which begins to be restricted to the eye primordia (Figure 3C). By stage 12, *Pt-Pax6.1* is expressed in specific subsets of cells in the developing brain, and the pattern continues to increase in complexity during the later stages of embryonic development (Figure 3D–F). *Pt-Pax6.1* and *Pt-so1* expression patterns do not overlap after stage 9.2 (Figure 3C–F), but two domains of *Pt-Pax6.1* expression appear to be immediately below (i.e., proximal to) the *Pt-so1* expression domains in the principal eye primordia at stage 13.2 (Figure 3F). These may represent brain structures, such as visual neuropils associated with the AMEs.

*Pt-Pax6.2* expression during stages 9.1 and 9.2 is similar to that of *Pt-Pax6.1*, although it has a bifurcated pattern, and appears to extend further towards the posterior edges of the head, where the secondary eye primordia are formed (Figure 3G,H). However, *Pt-Pax6.2* expression does not overlap with that of *Pt-so1* at these stages, being instead adjacent to it (Figure 3G,H). A similar pattern of *Pt-Pax6.2* expression is seen at stage 10.2, with the anterior being most domains adjacent to the principal eye primordia and the posterior being most domains adjacent to the secondary eye primordia (Figure 3I). By stage 12, the overall pattern of *Pt-Pax6.2* becomes restricted to a smaller subset of cells in the developing brain, with the largest domain immediately posterior to the principal eye primordia (Figure 3J). At stage 13.1, as the non-neurogenic ectoderm outgrows the developing brain, the principal eye primordia migrate just above the *Pt-Pax6.2* expression domains in the brain (Figure 3K). During the later stages of embryonic development, *Pt-Pax6.2* expression remains in the developing brain and does not overlap with *Pt-so1* expression in the eye primordia, although, similar to *Pt-Pax6.1*, some domains are immediately adjacent to the principal eye primordia, possibly in brain regions associated with the AMEs (Figure 3L).

### 3.2. Expression of an Eyegone Ortholog in the Developing Head of P. tepidariorum

*Pt-eyg* is mainly expressed in the labrum, prosomal appendages and opisthosomal organs (Supplementary Materials Figure S1). Expression is first observed at stage 10.1 in the tips of the chelicerae and as a single ring of expression in the pedipalps and walking legs, with faint expression already present in the labrum and opisthosomal organs (Supplementary Materials Figure S1A–A’’). At stage 11, a ring of expression is visible in the middle of the chelicerae, two additional fainter rings are visible in the pedipalps, and three additional rings form in the walking legs (Supplementary Materials Figure S1B,B’). During this stage, a small domain of expression is also present at the tips of the pedipalps and walking legs, and expression in the opisthosomal organs becomes stronger (Supplementary Figure S1B’,B’’). An additional expression domain was also detected in the dorsal tissue of opisthosomal segments (Supplementary Materials Figure S1B’’). By stage 13.1, expression appears to be stronger in the same spatial pattern (Supplementary Materials Figure S1C–C’’). We did not detect the expression of *Pt-eyg* in or adjacent to any eye primordia of *P. tepidariorum*.

### 3.3. Hh and Dpp Gene Expression in the Developing Head of P. tepidariorum

**Figure 4:**
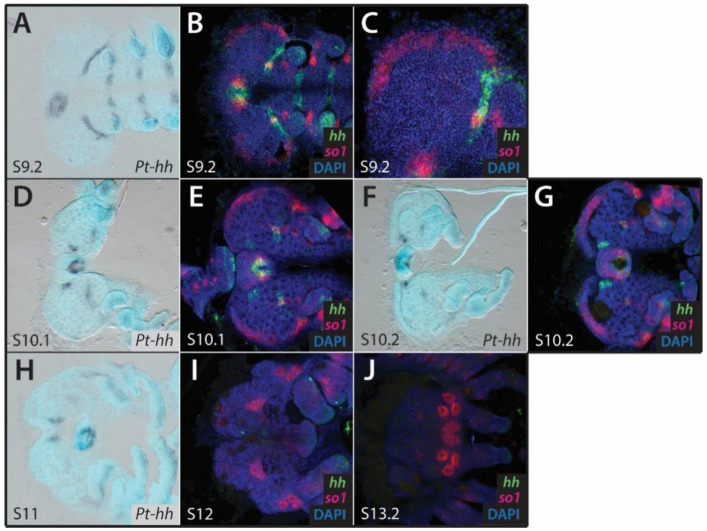
Expression of *hh* and *dpp* in the developing head of *Parasteatoda tepidariorum* embryos. Colorimetric ISH (**A**,**D**,**F**,**H**) and dFISH (**B**,**C**,**E**,**G**,**I**,**J**); co-stained with *Pt-so1* to visualize the eye primordia) showing *Pt-hh* expression in the developing head of *P. tepidariorum* embryos. Two stripes of *Pt-hh* expression are observed at stage 9.2 (**A**–**C**), near the posterior most border of *Pt-so1* expression in the eye primordia (**B**,**C**). These domains do not overlap *Pt-so1* expression (**C**) become increasingly fainter during stages 10.1 (**D**,**E**) and 10.2 (**F**,**G**), and are no longer visible at stage 11 (**H**). No expression of *Pt-hh* was observed from stage 12 onwards (**I**,J). Anterior is to the left in all images.

At stage 9.2, *Pt-hh* is expressed in the labrum and in two stripes in the neuroectoderm (Figure 4A). The latter domains extend from the center of the head to the posterior lateral edge of the head lobes, anterior to the developing chelicerae, and immediately adjacent to the posterior most region of *Pt-so1* expression (Figure 4A–C). This appears to be in a similar position to an observed stripe of *Pt-Pax6.2* expression (Figure 3G). The stripe domains of *Pt-hh* become much fainter during stages 10.1 and 10.2 (Figure 4D–G), and are no longer visible by stage 11 (Figure 4H). *Pt-hh* expression in the labrum is still observed at stages 10.1, 10.2, and 11, with two additional domains of expression in cell clusters on either side of the labrum (Figure 4D–H). No expression of *Pt-hh* was observed in the head from stage 12 onwards (Figure 4I,J).

We did not detect any domains of *Pt-dpp* expression in or near the eye primordia at any stage (Supplementary Materials Figure S2). *Pt-dpp* expression in the developing head is only observed in the labrum from stage 10.1 (Supplementary Materials Figure S2A) and becomes stronger during the subsequent stages (Supplementary Materials Figure S2B,C). Other domains of *Pt-dpp* expression were observed at the tips of all prosomal appendages and at the dorsal edge of the opisthosomal region (Supplementary Materials Figure S2).

### 3.4. Expression of an Atonal Ortholog Detected in the Developing Eyes of P. tepidariorum

**Figure 5:**
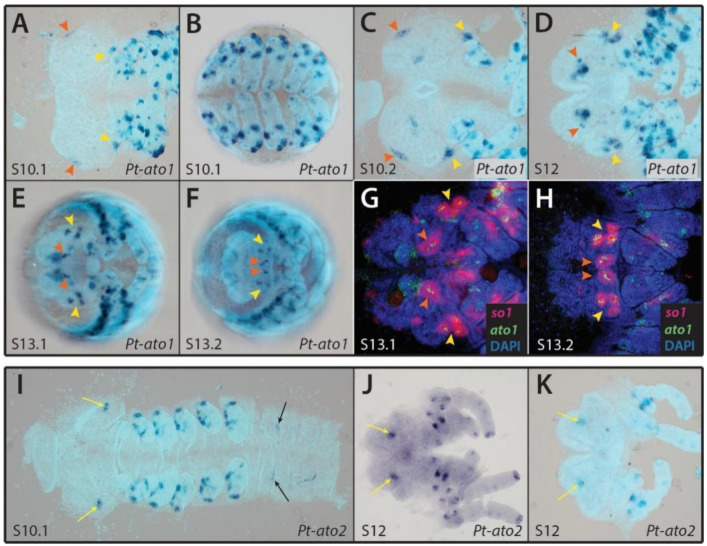
Expression patterns of *Parasteatoda tepidariorum atonal* genes. (**A**–**H**) Colorimetric ISH showing expression pattern of *Pt-ato1* in stage 10.1 (**A**,**B**), 10.2 (**C**), 11 (**D**), 12 (**E**) and 13.2 (**F**) embryos, and dFISH showing expression of *Pt-ato1* within *Pt-so1* expression domains in the developing eyes of stage 13.1 (**G**) and 13.2 (**H**) embryos. (**I**–**K**) Expression pattern of *Pt-ato2* in stage 10.1 (**I**) and 12 (**J**,**K**) embryos. Anterior is to the left in all images. In images (**A**,**C**,**D**,**J**,**K**), only the head region is shown. Embryos in (**A**,**C**,**D**) and (**G**–**K**) are flatmounted. Arrowheads indicate the principal (orange) and secondary (yellow) eye primordia. Arrows indicate the expression domains in the pre-cheliceral region (yellow) and in the second opisthosomal segment (black).

We identified two copies of *ato* in the genome of *P. tepidariorum*, *Pt-ato1* and *Pt-ato2* (Supplementary Materials Figure S3). Expression of *Pt-ato1* in the pre-cheliceral region is first visible at stage 10.1, in four small groups of cells that appear to be similar in position to the developing eye primordia (Figure 5A, arrowheads). This expression becomes stronger at stage 10.2, and two additional domains appear in the developing brain (Figure 5C). At stage 12, the expression domain in the principal eye primordia moves towards the center, and the expression domain in the secondary eye primordia subdivides into the expected three pairs of secondary eyes (Figure 5D, arrowheads). This pattern is maintained up to stage 13.2, when it becomes restricted to fewer cells within each developing eye (Figure 5E–H). *Pt-ato1* is also expressed in several clusters of cells in the developing prosomal appendages at stage 10.1, with an increasingly complex pattern in subsequent stages, that is likely associated with the developing peripheral nervous system (Figure 5A–F).

*Pt-ato2* is expressed in two small groups of cells in the pre-cheliceral region, although these appear to be located in the neuroectoderm (Figure 5I–K, yellow arrows). Both domains of expression are still present in the middle of the developing brain lobes at stage 12 (Figure 5J,K). *Pt-ato2* is also expressed in the prosomal appendages, in a similar pattern to that of *Pt-ato1*, although it seems to be expressed in fewer cells (Figure 5I–K). An additional expression domain can be seen in the forming limb buds of the second opisthosomal segment, possibly in the booklung primordia (Figure 5I, black arrows).

### 3.5. Wnt Gene Expression in the Developing Head of P. tepidariorum

To verify whether Wnt signaling could be involved in regulating eye development, we analyzed the expression patterns of Wnt genes in the developing head of *P. tepidariorum*, using dFISH with *Pt-so1* expression as a marker for the eye primordia. We focused on stages 10.2, when *Pt-so1* expression is restricted to the eye primordia, and 12, when the expression of *Pt-so1* in the secondary eye primordia separates into three distinct pairs corresponding to the secondary eyes.

**Figure 6:**
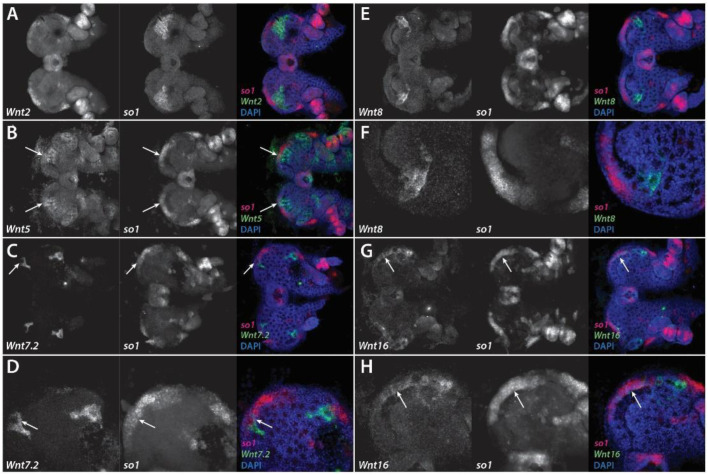
Wnt gene expression in the developing head of stage 10.2 *Parasteatoda tepidariorum* embryos. Expression of *Pt-Wnt2* (**A**), *Pt-Wnt5* (**B**), *Pt-Wnt7.2* (**C**,**D**), *Pt-Wnt8* (**E**,**F**), and *Pt-Wnt16* (**G**,**H**) in the developing head of stage 10.2 embryos. All embryos were co-stained for *Pt-so1* to visualize the developing eyes. Panels D, F, and H represent higher magnification images, focusing on one head lobe. Anterior is to the left in all images. White arrows indicate the regions where overlap of Wnt gene expression with *Pt-so1* expression was detected.

**Figure 7:**
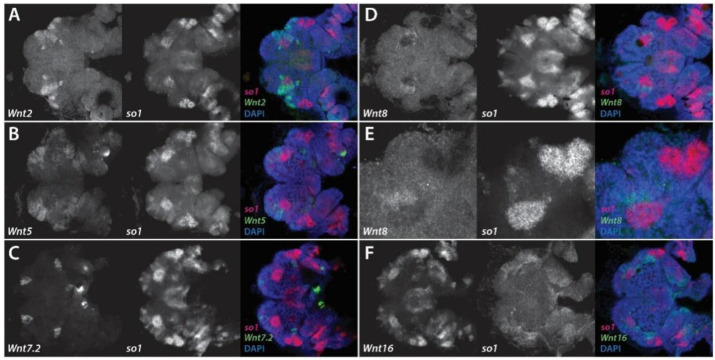
Wnt gene expression in the developing head of stage 12 *Parasteatoda tepidariorum* embryos. Expression of *Pt-Wnt2* (**A**), *Pt-Wnt5* (**B**), *Pt-Wnt7.2* (**C**), *Pt-Wnt8* (**D**,**E**) and *Pt-Wnt16* (**F**) in the developing head of stage 12 embryos. All embryos were co-stained for *Pt-so1* to visualize the developing eyes. Panel E represents a higher magnification image, focusing on one head lobe. Anterior is to the left in all images.

At stage 10.2, *Pt-Wnt2* is expressed in two large clusters of cells, extending from the center of the head up towards the lateral rim of each head lobe, where the region of expression is at its widest (Figure 6A). This expression domain does not seem to extend into the non-neurogenic ectoderm, and is posteriorly adjacent to *Pt-so1* expression in the principal eye primordia (Figure 6A). By stage 12, *Pt-Wnt2* expression appears to surround *Pt-so1* expression in the principal eye primordia, which have migrated to a more central location in the head, on the lateral and anterior sides (Figure 7A). *Pt-Wnt2* expression on the lateral margins of the head extends to just above the PME primordia (Figure 7A).

*Pt-Wnt5* is expressed in four large domains, two on each head lobe of stage 10.2 embryos, which extend into the non-neurogenic region between the principal and secondary eye primordia domains of *Pt-so1* expression (Figure 6B). *Pt-Wnt5* expression appears to surround both principal and secondary eye primordia at stage 10.2, but partially overlaps with the anterior-most region of the former (Figure 6B). Intriguingly, this particular domain of *Pt-so1* expression then becomes restricted to a smaller group of cells, suggesting that *Pt-so1* expression may be lost where it overlaps with *Pt-Wnt5* expression. At stage 12, *Pt-Wnt5* expression appears to be restricted to four domains: in the non-neurogenic region anterior to the principal eye primordia, and in the neurogenic ectoderm region between the principal and the secondary eye primordia (Figure 7B). The anterior-most domain of *Pt-Wnt5* expression appears to partially overlap *Pt-Wnt2* expression in this region (Figure 7A,B).

*Pt-Wnt7.2* expression is restricted to four small clusters of cells, which are adjacent to the four *Pt-so1* expression domains in the eye primordia at stage 10.2 (Figure 6C). It appears that *Pt-Wnt7.2* expression also slightly overlaps with *Pt-so1* expression in the principal eye primordia, in the same area as *Pt-Wnt5* at stage 10.2 (Figure 6B–D). At stage 12, *Pt-Wnt7.2* expression is still restricted to these four domains: two at the anterior rim above the principal eye primordia, and two medial to the secondary eye primordia (Figure 7C). The former appears to be immediately adjacent to the *Pt-Wnt5* expression domain at the rim of the pre-cheliceral region (Figure 7B,C).

At stage 10.2, *Pt-Wnt8* is expressed in two small groups of cells, which sit immediately adjacent to the lateral-most border of *Pt-so1* expression in the principal eye primordia (Figure 6E). This does not appear to overlap with *Pt-so1* expression (Figure 6F). Expression of *Pt-Wnt8* becomes fainter at stage 12, when it surrounds *Pt-so1* expression in the principal eye primordia on the lateral and anterior sides (Figure 7D,E).

Lastly, *Pt-Wnt16* expression appears to be restricted to the lateral rims of the developing head at stage 10.2 (Figure 6G). Expression appears to be immediately anterior to the secondary eye primordia, though it also seems to extend into the *Pt-so1* expression domain in the principal eye primordia (Figure 6G,H). However, *Pt-Wnt16* expression does not completely overlap with *Pt-so1* expression in this domain (Figure 6H). *Pt-Wnt16* expression at stage 12 appears to be restricted to the same area of the developing head, seemingly strongest immediately anterior to the secondary eye primordia, and no longer overlaps with *Pt-so1* expression in the principal eye primordia (Figure 7F).

## 4. Discussion

### 4.1. Initiation of Spider Eye Development: No Apparent Role for Pax6 or Other Candidates

A previous study of RDG expression in *P. tepidariorum* did not detect the expression of either *Pax6* ortholog in the developing eyes [12]. However, the authors proposed that *Pax6* might be required during the earlier establishment of the eye primordia, in line with its role in *Drosophila melanogaster*, as it is expressed in the anterior rim of the germ band during earlier embryonic stages [12]. We did not detect the expression of either *Pax6* ortholog in the developing eyes (i.e., overlapping with *Pt-so1*) of *P. tepidariorum* from stage 10.1 onwards. Instead, we detected the expression of both *Pax6* orthologues in the neuroectoderm, including regions of the developing brain that are adjacent to or underlying the AMEs.

We did observe a small overlap in expression between *Pt-Pax6.1* and *Pt-so1* at stage 9.2 when *Pt-so1* expression first appears, which is earlier than previously described [12]. However, this area of overlap subsequently loses *Pt-so1* expression, when the eye primordia for the principal and secondary eyes are established. Therefore, *Pt-Pax6.1* could instead be restricting the early expression domains of *Pt-so1* to each eye primordium, in contrast to its role in other taxa.

Overall, our results suggest that *Pax6* expression is not necessary for the activation of *Pt*-*so1* expression in *P*. *tepidariorum*. In the horseshoe crab *Limulus polyphemus*, the only other chelicerate species where *Pax6* expression has been analyzed in relation to eye development, the establishment of eye primordia also appears to be independent of *Pax6* expression [47]. In *C*. *salei*, the *Pax6* gene orthologous to *Pt-Pax6.1* is apparently expressed in the AMEs, but this was detected at a late stage of embryogenesis, and is therefore unlikely to indicate an upstream regulatory role [13]. We detected *Pax6* expression near the AMEs at a similar stage of development in *P. tepidariorum*, but we believe that this is attributable to expression in the visual neuropil beneath the eye primordia [48,49]. The expression patterns that we detected in earlier stages of the neuroectoderm in *P. tepidariorum* are also similar to those reported for *C. salei* [13]. We tentatively suggest that *Pax6* may be expressed in the visual neuropils of spider AMEs, but not in the eyes themselves.

We cannot completely exclude the possibility that *Pax6* has a role to play in either spiders or horseshoe crabs. Only one copy of *Pax6* was identified and characterized in *L. polyphemus*, but the horseshoe crab lineage is believed to have undergone at least two rounds of WGD [50,51,52]. Therefore, additional unannotated copies of *Pax6* might be present in the genomes of horseshoe crabs and involved in their eye development. However, involvement seems unlikely in spiders, given that the *Pax6* genes in *P. tepidariorum* are almost completely absent from the eye primordia. The only small and brief overlap between *Pt-Pax6.1* and *Pt-so1* could instead indicate that *Pt-Pax6.1* restricts the early expression domains of *Pt-so1* to each eye primordium; however, this remains speculative.

Given the apparent absence of *Pax6* at the top of the RDG network in spiders, we also characterized the expression patterns of *Pt-eyg*, an ortholog of the *Drosophila* genes *eyg* and *toe*. These are known to be required for eye development in *D. melanogaster,* and can compensate for *Pax6* activity, and suppress *wg* [34,35]. However, we did not detect any expression of *Pt-eyg* in the developing head of *P. tepidariorum*. The molecular factor(s) responsible for initiating eye development in this spider therefore remain elusive.

### 4.2. Roles of Dpp and Hh Are Not Conserved between Spiders and Insects

*Dpp* and *hh* are essential in the regulation of RDGs in *D. melanogaster*. *Dpp* is required for the maintenance of the morphogenetic furrow, where it activates the expression of *so*, *eya*, and *dac* [18]. *hh* activates the expression of *so* and *eya* in the compound eye and *eya* and *otd* in the ocelli [21,22].

We did not detect any expression of *Pt-dpp* in the developing head of *P. tepidariorum*, suggesting that eye development is independent of this signaling pathway. This was unexpected, since the role of the Dpp/BMP signaling pathway activating RDG expression appears to be conserved in both *D. melanogaster* and vertebrates [19,26,53,54]. It is possible that the Dpp/BMP signaling pathway was independently co-opted to eye development in these two lineages, but the loss of Dpp involvement in spiders would be more parsimonious. 

Two domains of *Pt-hh* expression were detected in the head primordia of stage 9.2 embryos, at the onset of *Pt-so1* expression. Although these domains are in close proximity to *Pt-so1* expression, they do not overlap, suggesting that *Pt-hh* does not play a role in activating RDG expression. They also do not correspond to the patterns of *otd2* expression previously described in the principal eye primordia by Schomburg et al. [12], despite *hh* activating *otd* in the ocelli of *D. melanogaster* [22]. Schomburg et al. described a stripe of *otd1* expression similar to the stripes of *hh* that we report here, which appeared in stage 10 [12]. Given its position, the stripe of *Pt-hh* could instead have a role restricting *Pt-so1* expression at its posterior-most border. Although this is at odds with the role of *hh* in *D. melanogaster*, it is reminiscent of *Sonic hedgehog* (*Shh*) in vertebrates: *Shh* suppresses *Pax6* medially, splitting the growing eye field into two, while Wnt signaling suppresses eye development laterally and ventrally [26,53,54,55]. Indeed, the *hh* expression that we observe in *P. tepidariorum* also appears to complement Wnt gene expression surrounding the eye primordia anteriorly. This could represent either conservation or convergence between vertebrate and arachnid RDG regulation. Although the expressions of the two genes were not studied simultaneously, it appears that *Pt-hh* and *Pt-Pax6.2* expression could overlap in the neurogenic ectoderm at stages 9.2 and 10.2. A functional relationship may therefore persist between them, but this is apparently unrelated to eye development.

### 4.3. Atonal Orthologs Could Be Responsible for Photoreceptor Cell Fate in P. tepidariorum

One of the main downstream targets of the RDG network in *D. melanogaster* is the proneural gene *ato*, which plays an essential role in the differentiation of photoreceptor neurons [23]. Similar regulation is found in vertebrate eye development, with *Pax6* directly regulating *Ath5*, an ortholog of *ato* required to specify a subset of neurons in the retina [56]. This suggests a conserved role for *ato* across varying phylogenetic distances.

We identified two copies of *ato* in the genome of *P. tepidariorum*, one of which is expressed in both principal and secondary eye primordia as early as stage 10.1 (Figure 5A). *Pt-ato1* expression appears to become restricted to a subset of cells in each eye primordium at later stages of development (Figure 5E–H). This is evocative of *ato* expression in the eye disc of *D. melanogaster*, where it is at first broadly expressed in a proneural domain and subsequently becomes restricted to a single primary neuronal precursor in each developing ommatidium, corresponding to the R8 photoreceptor cell [23]. Thus, it is plausible that *Pt-ato1* performs a similar function in *P. tepidariorum*, first specifying neuronal fate within the eye primordia before being restricted to a single neuronal cell type. *ato*’s position as a downstream target of the RDG network and its associated role in specifying neuronal fate in the eye appears to be conserved in most other animals studied so far [1,5,23,56]. Although both *Pt-ato1* and *Pt-Pax6.1* expression overlap with *Pt-so1* expression in the principal eye primordia in later stages of development, we believe that *Pt-Pax6.1* underlies the eye primordia, so this is not likely to reflect a functional relationship between them. Furthermore, *Pt-ato1* expression begins in the principal eye primordia much earlier than the apparent *Pt-Pax6.2*.

However, these results are inconsistent with observations in *C. salei*, wherein *ato* expression was not detected in the developing eyes [13]. Furthermore, an analysis of the expression of a single horseshoe crab *ato* ortholog found no evidence for its role in eye development [47], although it remains possible that other copies of *ato* present in the genome of *L. polyphemus* may be involved. Nevertheless, the apparent absence of *ato* expression in the developing eyes of *C. salei* is puzzling.

### 4.4. A Conserved Role for Wnt Signalling in Spider Eye Development?

**Figure 8:**
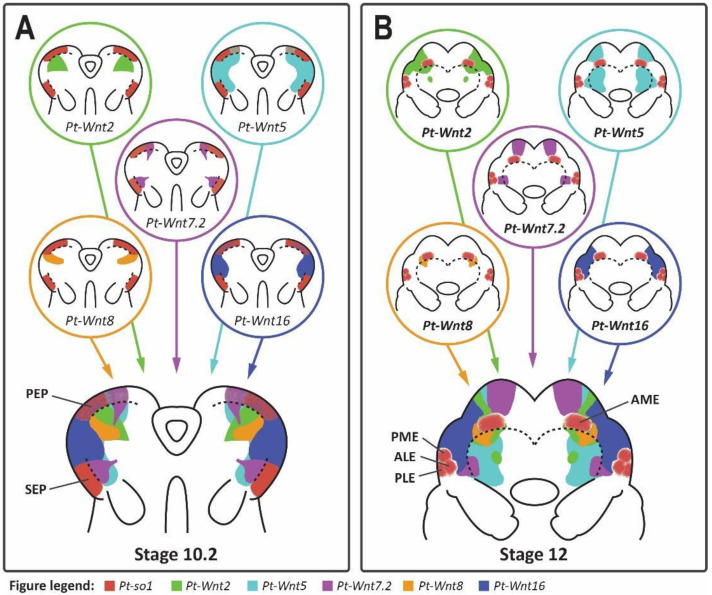
Summary of Wnt gene expression in the developing head of *Parasteatoda tepidariorum*. Summary of the expression patterns of *Pt-Wnt2*, *Pt-Wnt5*, *Pt-Wnt7.2*, *Pt-Wnt8* and *Pt-Wnt16* in the developing head of stage 10.2 (**A**) and stage 12 (**B**) *P. tepidariorum* embryos, in relation to *Pt-so1* expression. Expression pattern of each individual Wnt gene (top) as well as the sum of all expression domains (bottom) is shown for both stages. PEP, principal eye primordia; SEP, secondary eye primordia; AME, anterior median eye; ALE, anterior lateral eye; PME, posterior median eye; PLE, posterior lateral eye.

We found that Wnt expression in the developing head of *P. tepidariorum* is observed mostly adjacent to the eye primordia. This is especially true in the case of the secondary eye primordia, which are surrounded by the combined expression domains of *Pt-Wnt2*, *Pt-Wnt5*, *Pt-Wnt7.2*, and *Pt-Wnt16* (Figure 8). This pattern resembles that of Wnt expression during both insect compound eye and vertebrate camera eye development, where Wnt signaling restricts the expression of the RDGs to their appropriate domains [20,53]. Thus, it is plausible to infer that Wnt signaling plays a role in restricting the expression of retinal determination genes, such as *Pt-so1*, and thus the extent of the retinal field, in *P. tepidariorum*. Moreover, this appears to be achieved by the combined effect of multiple Wnt ligands, similar to vertebrates [57]. 

We observed a slightly different scenario in the principal eye primordia. Here, *Pt-so1* expression is contiguous to *Pt-Wnt2* and *Pt-Wnt8* expression, although it is partially overlapped by the expression domains of *Pt-Wnt5*, *Pt-Wnt7.2,* and *Pt-Wnt16* during the early stages of eye development. This is reminiscent of the developing ocelli in *D. melanogaster* (Figure 1) [22], which are thought to be homologous to the principal eyes [1]. The ocellus primordia are established in a region of the eye disc where *wg* is expressed, and in contrast to the compound eyes, this acts via *otd* and *toy* (a *Pax6* gene) to activate the expression of *eya* and *so,* respectively (Figure 1) [22]. The principal eye primordia of *P. tepidariorum* also exhibit this distinctive combination of *Pt-otd2*, *Pt-so1* and *Pt-eya* expression [12]. Therefore, the gene network regulating the establishment of the principal eye primordia in *P. tepidariorum* appears to be conserved with respect to the ocellus primordia in *D. melanogaster*; however, the role played by *wg* in the latter may be carried by the combined effect of multiple Wnt ligands in the former.

**Figure 9:**
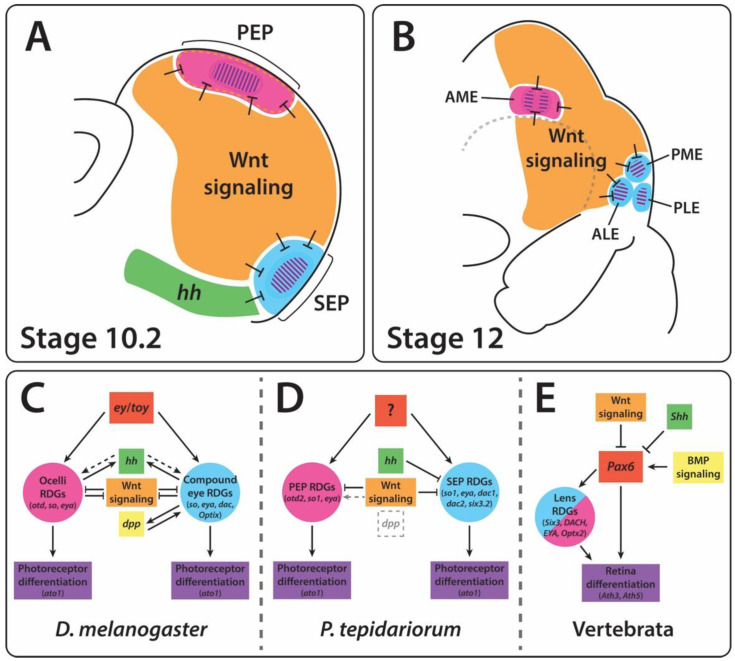
Proposed gene regulatory network controlling eye development in *Parasteatoda tepidariorum* and comparison with insects and vertebrate RDGNs. (**A**) Summary of gene expression reported in this study that may regulate eye development, shown in a stage 10.2 embryo. Wnt signaling and *hh* suppress the expression of RDGs in the tissue surrounding the developing eye primordia. *Atonal* expression (purple hatching) is present in a subpopulation of cells within the eye primordia. Some Wnt expression was observed overlapping the PEP (dashed lines). (**B**) Summary of gene expression in a stage 12 embryo. Wnt signaling suppresses RDG expression in the eye primordia, and *atonal* expression is present in a subpopulation of cells at the centre of each eye. (**C**–**E**) Proposed networks regulating eye development in *Drosophila melanogaster, P. tepidariorum* and vertebrates. (**C**) In *D. melanogaster*, *ey/toy* initiate RGD networks in the ocellus (pink) and compound eye (blue) primordia. These are suppressed by Wnt signaling and reciprocally activated by *hh* and *dpp*. (**D**) In *P. tepidariorum,* Wnt, and *hh* suppress RDGs in the principal (pink) and secondary (blue) eye primordia, while Dpp is absent, and the upstream initiation of RDGs remains unknown. Wnt signaling could potentially activate RDGs during early eye development (grey dashed arrow). (**E**) In vertebrates, *Pax6* initiates RDGs in the eye primordia (only one type, pink/blue), itself regulated by Wnt, *Shh* and BMP/Dpp signaling.

In summary, our results suggest that Wnt signaling may play a substantial and conserved role in regulating the eye development of spiders, much like its role in vertebrate and insect eye development, possibly restricting RDG expression to each eye primordium. Theoretically, differences in the size and position of spider eyes could be achieved through changes in Wnt gene expression patterns. Consistent with this, evidence of divergence in Wnt gene expression was found in a recent study of three species of spiders, including *P. tepidariorum* [58]. Furthermore, the regulation of each eye type appears to be mechanistically different, reminiscent of *D. melanogaster* (Figure 9). Nevertheless, further investigation is necessary to better understand the role of Wnt signaling during spider eye development, and the functional analysis of Wnt genes, with respect to their function in the developing head of *P. tepidariorum,* will be crucial for the validation of this hypothesis.

## 5. Conclusions

Although eyes likely have many evolutionary origins, researchers have consistently found developmental mechanisms that link the eyes of vertebrates, insects, and other groups, and suggest conserved roles across substantial phylogenetic distances. In both *D. melanogaster* and vertebrate models, the retinal determination cascade appears to be initiated by *Pax6*, culminates in *ato*-mediated photoreceptor differentiation, and is spatially constrained by Wnt signaling. In *D. melanogaster,* the primordia of the two eye types, compound eyes, and ocelli, express different combinations of RDGs (Figure 9). Previous studies have demonstrated that the components of the central cascade are conserved in spiders, including the different suites of RDGs in the principal and secondary eyes. We report the first evidence that the roles of *ato* and Wnt signaling may also be consistent across arthropods, potentially including differentiation between eye types. However, support for *Pax6* as the ‘master regulator’ in spiders remains scant, and no obvious candidates have emerged for the initiation of eye determination (Figure 9). We also found no evidence for the involvement of Dpp/BMP signaling, in contrast to both insect and vertebrate eye development. *hh* may suppress eye determination in *P. tepidariorum*, unlike *D. melanogaster*, but is aligned with the role of *Shh* in vertebrates. 

## Data Availability

The data presented in this study are available in the figures and Supplementary Materials.

## Supplementary Materials

The following supporting information can be downloaded at: https://www.mdpi.com/article/10.3390/cells11040631/s1, Figure S1: Expression of *eyegone* in *Parasteatoda tepidariorum* embryos; Figure S2: Expression of *dpp* in *Parasteatoda tepidariorum* embryos; Figure S3: Maximum likelihood phylogeny of arthropod *ato* and *twist* genes, including orthologs identified in *Parasteatoda tepidariorum;* Table S1: List of primers used to synthesis ISH probes.
