# Supplementary material for: Regulation of Eye Determination and Regionalization in the Spider Parasteatoda tepidariorum
Source: Cells. 2022 Feb 11;11(4):631. doi: 10.3390/cells11040631 (PMC8870698; doi:10.3390/cells11040631)
Supplement: Supplementary file 1 [file cells-11-00631-s001.zip › cells-1544364-supplementary.pdf]

Supplementary Materials:

Regulation of Eye Determination and Regionalization in the Spider *Parasteatoda tepidariorum*

Luis Baudouin-Gonzalez [1,*], Amber Harper [2], Alistair P. McGregor [2,3] and Lauren Sumner-Rooney [4]

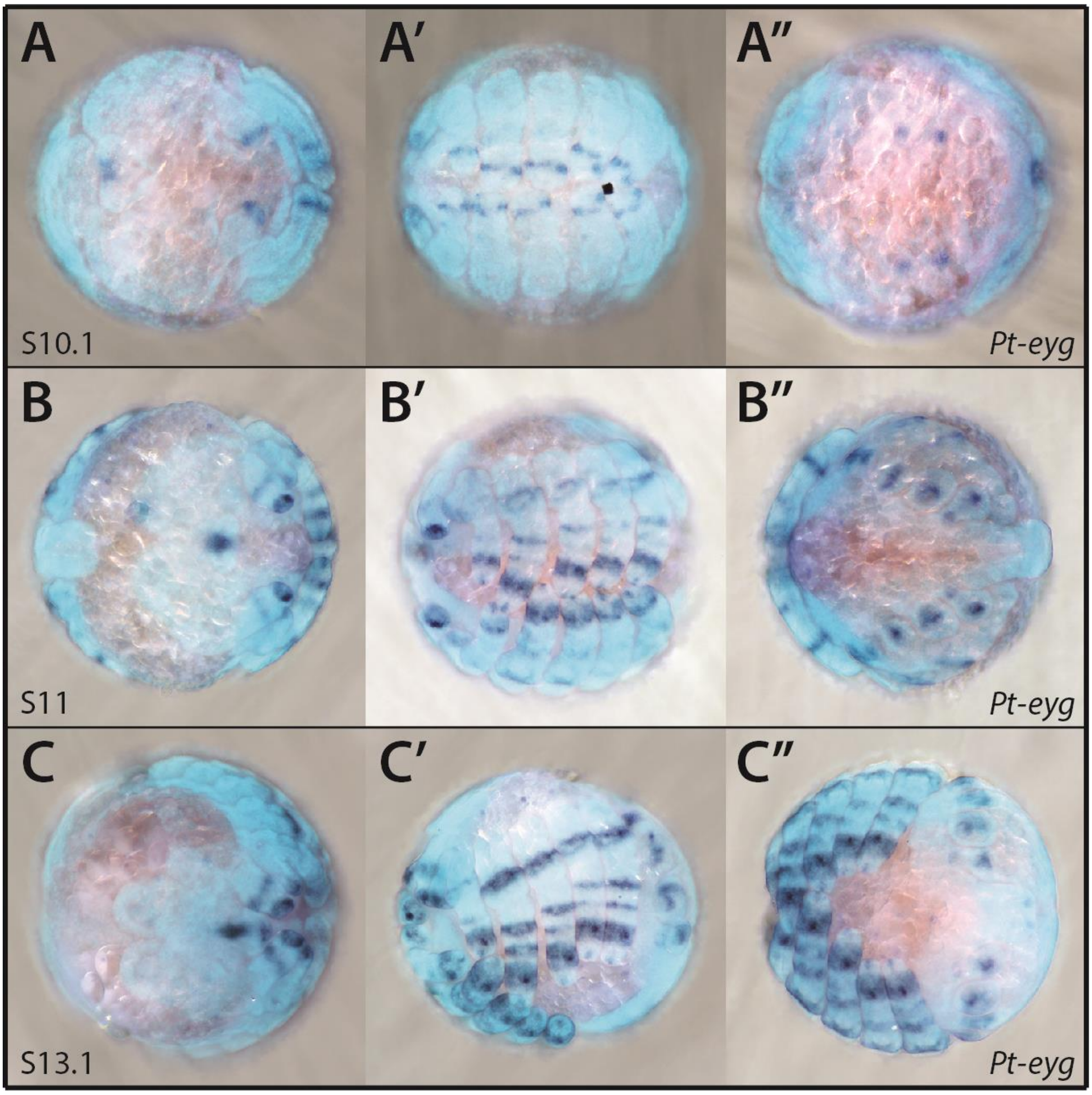

Figure S1. Expression of *eyegone* in *Parasteatoda tepidariorum* embryos. Colorimetric ISH showing *Pt-eyg* expression in stage 10.1 (A-A′′), 11 (B-B′′) and 13.1 (C-C′′). No expression was detected in or around the developing eye primordia.

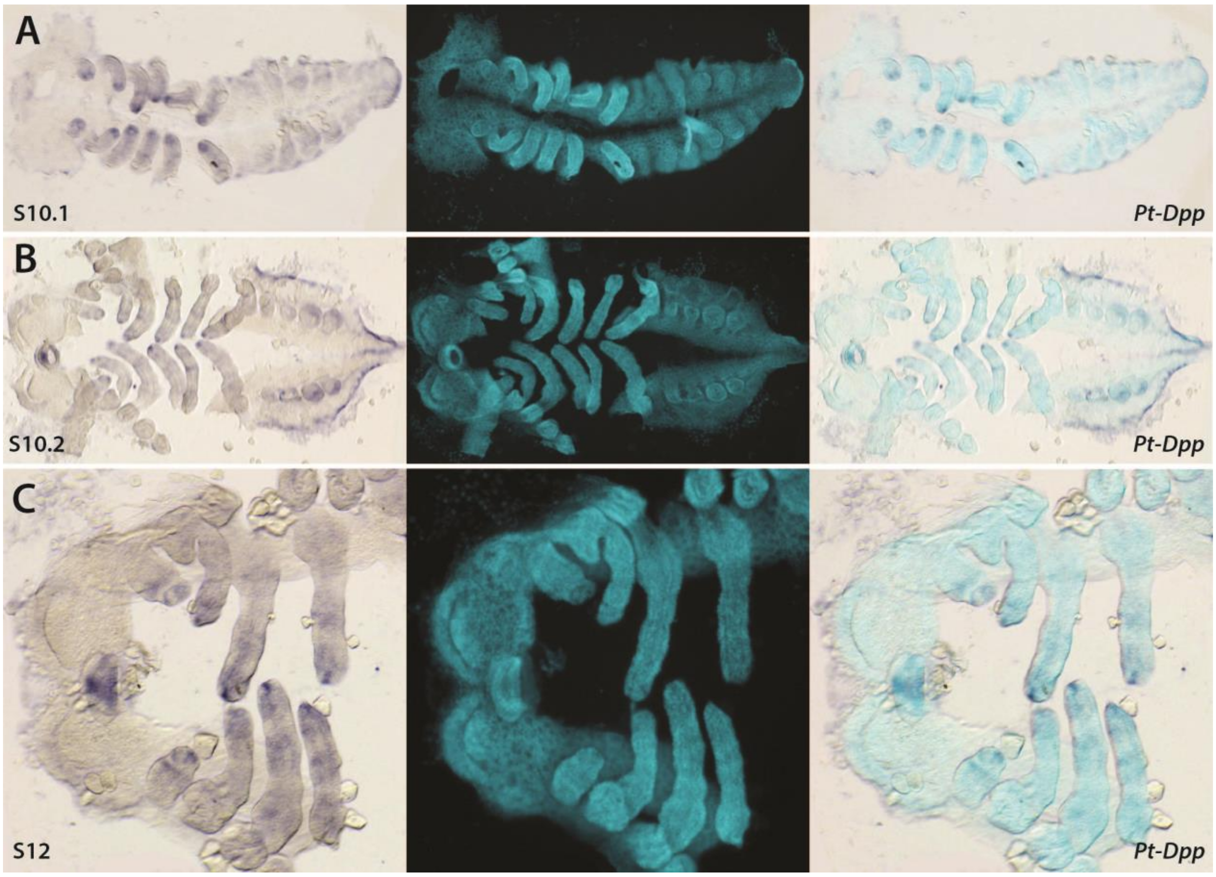

Figure S2. Expression of *dpp* in *Parasteatoda tepidariorum* embryos. Colorimetric ISH showing *Pt-dpp* expression in stage 10.1 (A-A''), 10.2 (B-B'') and 12 (C-C''). No expression was detected in or around the developing eye primordia.

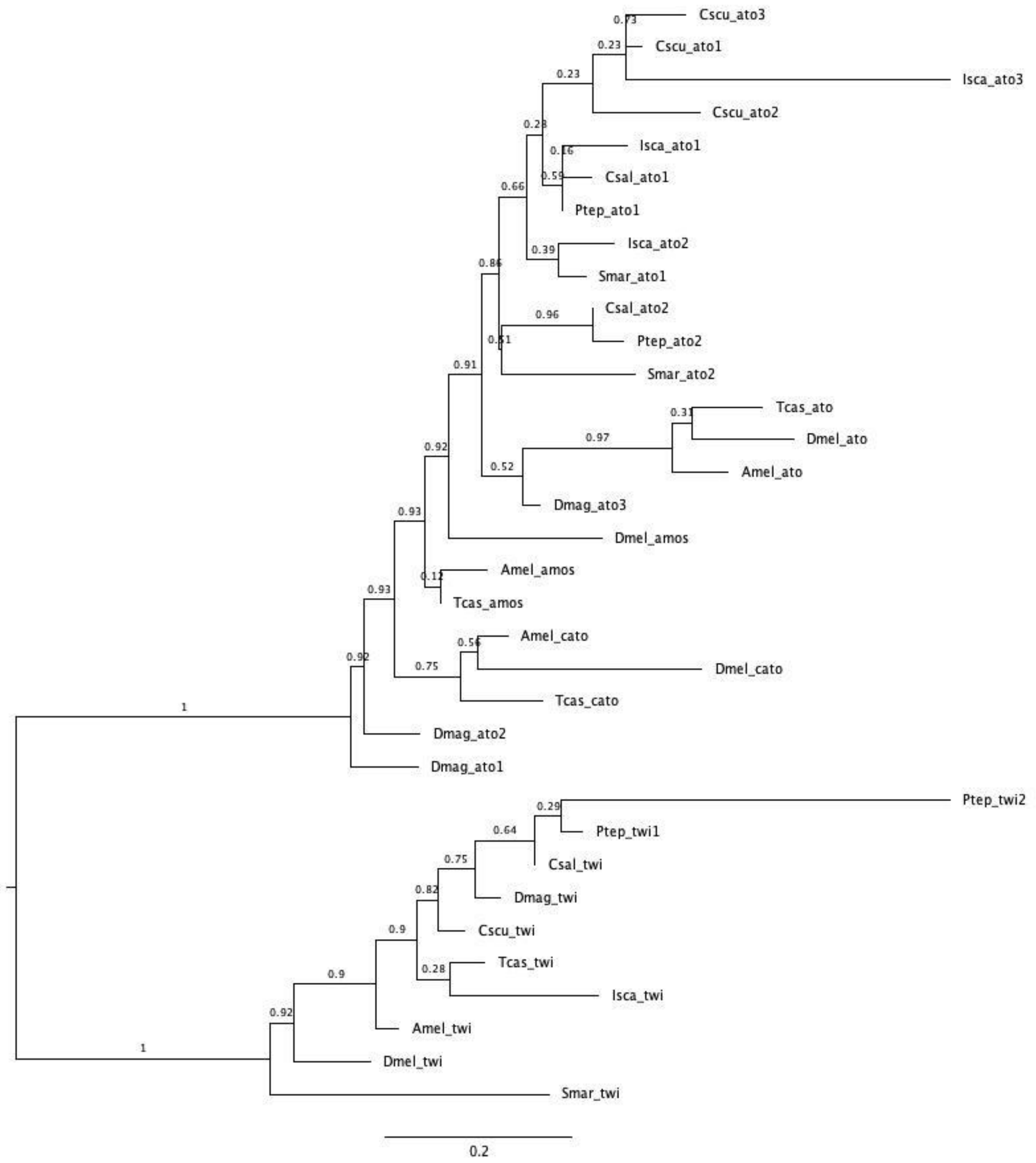

Figure S3. Maximum likelihood phylogeny of arthropod *ato* and *twist* genes, including orthologs identified in *Parasteatoda tepidariorum*. Two copies of *ato* and two potential copies of *twist* were recovered from *P. tepidariorum*.
